# Genetic liability for anxiety and treatment response to the monoamine stabilizer OSU6162 in alcohol dependence: a retrospective secondary analysis

**DOI:** 10.1007/s43440-025-00707-8

**Published:** 2025-03-12

**Authors:** Mun-Gwan Hong, Lotfi Khemiri, Joar Guterstam, Johan Franck, Nitya Jayaram-Lindström, Philippe A. Melas

**Affiliations:** 1 Science for Life Laboratory, Department of Biochemistry and Biophysics, National Bioinformatics Infrastructure Sweden, Stockholm University, Stockholm, 17121 Sweden; 2 Center for Psychiatry Research, Department of Clinical Neuroscience, Karolinska Institutet & Stockholm Health Care Services, Stockholm, 11364 Sweden; 3 L8:01, Karolinska University Hospital, Stockholm, 17176 Sweden

**Keywords:** Alcohol use disorder, AUD, OSU, PNU-96391, Polygenic risk scores, Pharmacogenetics

## Abstract

**Background:**

OSU6162, a monoamine stabilizer, has demonstrated efficacy in reducing alcohol and anxiety-related behaviors in preclinical settings. In a previous randomized, double-blind, placebo-controlled trial involving patients with alcohol dependence (AD), OSU6162 significantly reduced craving for alcohol but did not alter drinking behaviors. This retrospective secondary analysis explores whether genetic predispositions related to AD and associated traits might influence the response to OSU6162 treatment in original trial participants.

**Methods:**

Polygenic risk scores (PRSs) were calculated for 48 AD patients using PRSice-2 and genome-wide association study (GWAS) data for (i) alcohol use disorder and alcohol consumption, (ii) problematic alcohol use, (iii) drinks per week, (iv) major depression, and (v) anxiety (case-control comparisons and quantitative anxiety factor scores). Linear regression analyses, adjusted for population stratification, assessed interaction effects between PRSs and treatment type (OSU6162 or placebo) on various clinical outcomes.

**Results:**

Significant interactions were found between treatment type and anxiety factor score PRS at the genome-wide significance threshold. In the OSU6162-treated group, a higher anxiety PRS was associated with reductions in the number of drinks consumed (FDR = 0.0017), percentage of heavy drinking days (FDR = 0.0060), and percentage of drinking days (FDR = 0.0017), with a trend toward reduced blood phosphatidylethanol (PEth) levels (FDR = 0.068). These associations were absent in the placebo group.

**Conclusions:**

These preliminary findings suggest that anxiety PRS may help predict response to OSU6162 treatment in AD. Further research with larger cohorts and more comprehensive genetic data is needed to confirm these results and advance personalized medicine approaches for alcohol use disorder.

**Supplementary Information:**

The online version contains supplementary material available at 10.1007/s43440-025-00707-8.

## Introduction

Alcohol dependence (AD) is a complex disorder characterized by dysregulated dopaminergic and serotonergic brain systems, which are crucial in modulating reward, craving, and cognitive functions [1, 2]. AD is also often associated with co-occurring anxiety and depressive disorders, further complicating the clinical picture [3]. These comorbid conditions share underlying familial liabilities with substance misuse [4] and have been linked to disturbances in monoamine neurotransmitter signaling [5–7]. Despite AD presenting a significant global health burden, a substantial number of affected individuals do not seek treatment, and an even smaller proportion receives FDA/EMA-approved pharmacotherapies [8–11]. Notably, two of the most highly regarded AD pharmacotherapies—naltrexone and acamprosate—yield suboptimal responses in approximately 40 to 70% of patients [12–14]. A recent pharmacogenomics study highlighted the association of two intergenic single nucleotide polymorphisms (SNPs) with specific treatment outcomes for these medications [15]. The concept of precision medicine — tailoring treatments based on individual genetic and molecular profiles — provided the rationale for the present study’s hypothesis that genetic predispositions might reveal who would benefit most from (-)-OSU6162 (OSU6162), also known as PNU-96,391, a monoamine stabilizer showing promising preclinical and clinical results in AD settings [16–21].

OSU6162 stabilizes dopaminergic and serotonergic signaling pathways by acting as a neutral antagonist or a weak partial agonist at dopamine D2 and serotonin 5-HT2A receptors [22–25]. Its documented efficacy in normalizing psychomotor activity and striatal dopaminergic function [26, 27] formed the basis for its application in disorders marked by dopaminergic dysregulation, such as AD. Preclinical studies using the intermittent access to 20% ethanol (IA20E) two-bottle-choice drinking model in long-term drinking rats have shown that OSU6162 (consistently at 30 mg/kg) reduces voluntary alcohol consumption, withdrawal symptoms, and the reinstatement of alcohol seeking [16, 17], alongside mitigating anxiety-like behaviors [18] and correcting downregulated dopamine output in the nucleus accumbens [19]. The efficacy of OSU6162 on drinking and craving has also been evaluated in a Phase II human study with AD individuals [20]. The treatment was safe, well-tolerated, and notably reduced priming-induced craving and the subjective liking of alcohol. Additionally, OSU6162 improved certain cognitive functions, including future planning, verbal divergent thinking, and emotional recognition speed [21]. Despite these promising findings, the study found no evidence of any treatment effects on alcohol intake [20].

In the present retrospective secondary analysis, we aimed to investigate whether genetic predispositions, particularly related to AD and the comorbid disorders of anxiety and depression, may influence therapeutic responses to OSU6162 treatment. By leveraging genome-wide association study (GWAS) data to calculate polygenic risk scores (PRSs) for alcohol use disorder, problematic alcohol use, alcohol consumption, major depressive disorder, and anxiety, the aim was to uncover putative genetic underpinnings of treatment response. This approach could benefit personalized medicine efforts in AD treatment, particularly when utilizing monoamine stabilizers like OSU6162, which could be tailored to individual genetic profiles.

## Materials and methods

### Study design and participants

This retrospective study builds upon a randomized, double-blind, placebo-controlled trial conducted between 2012 and 2013 at the Stockholm Centre for Dependency Disorders, Stockholm, Sweden, which investigated the effects of OSU6162 in individuals with alcohol dependence (AD) [20]. A total of 56 AD participants, aged 20–55 years, were randomized to receive either OSU6162 (*N* = 28) or placebo (*N* = 28) for 14 days. OSU6162 was administered following a stepwise dosing regimen: 10 mg twice daily (Days 1–5), 15 mg twice daily (Days 6–10), and 30 mg twice daily (Days 11–14). Participants were predominantly of European ancestry (85–90%), based on clinical impressions and personal communication with two study M.D.s. The gender distribution was nearly equal, with 30 males and 26 females. Eight participants (four from each treatment group) were excluded due to reasons such as relapse, noncompliance with study procedures, or testing positive for opiates. Data from the remaining 48 participants were included in the polygenic risk score (PRS) analyses. For full details on study methods and participant characteristics, see [20]. In brief, the study included three follow-up visits within the 14-day treatment period and a laboratory-based alcohol craving test session on day 15. Follow-up visits encompassed electrocardiogram (ECG), blood and urine sample collection, medication dispensing, breathalyzer tests, and self-reported drinking, mood, and adverse events. Participants met DSM-IV criteria for alcohol dependence, reported at least 45 heavy drinking days (HDD) in the preceding 90 days, and abstained from alcohol for 4 to 14 days before inclusion, confirmed by Timeline Follow Back (TLFB) interview [28] and breathalyzer. Exclusion criteria included other substance use disorders (except nicotine), schizophrenia, bipolar disorder, major depression, and significant cardiac or ECG abnormalities. The study was conducted in accordance with Good Clinical Practice and the Declaration of Helsinki, with approval from the Regional Ethics Committee of Stockholm (Dnr 2011/1707-31/4) and the Swedish Medical Products Agency. It was registered in the European Clinical Trials Database (EudraCT; 2011-003133-34) and all participants provided written informed consent.

### Clinical measures and alcohol craving test sessions

Mood and craving were assessed using the Montgomery-Åsberg Depression Self-Rating Scale (MADRS-S) [29] and the Penn Alcohol Craving Scale (PACS) [30], respectively. Alcohol consumption was quantified through changes in percent HDD, percent drinking days, and phosphatidylethanol (PEth) serum levels, along with the number of drinks, and percentage of both drinking days and HDD during the 14-day treatment period. On day 15, laboratory-based alcohol craving test sessions were conducted based on Hammarberg et al. [31], involving three sessions triggered by alcohol-specific cues, neutral stimuli, and a priming dose of alcohol. Craving was evaluated using the shortened Swedish version of the Desire for Alcohol Questionnaire (Short-DAQ) [32] and the Visual Analog Scale (VAS).

### GWAS data and quality control steps

Patient genotypes (*N* = 48) were determined using the Illumina Infinium Global Screening Array-24 v2.0 (Illumina Inc., San Diego, CA, USA). An additional 357 samples from different substance use projects were included in the genotyping array to optimize the use of the assay, forming a collective ‘target sample’ of 405 individuals. Post-quality control (QC) processing retained 547,984 of the original 730,059 genetic variants included in the assay. The QC filtered out variants with over 10% missing genotypes, minor allele count of one or none, or significant Hardy-Weinberg equilibrium violation (*P* < 1e-10).

### Polygenic risk scores

PRSs were derived from seven base datasets, including GWASs for alcohol use disorder [33], alcohol consumption based on Alcohol Use Disorders Identification Test-Consumption (AUDIT-C) scores [33], problematic alcohol use [34], drinks per week [35], major depression [36], and two approaches to anxiety disorder phenotyping (case-control comparisons and quantitative anxiety factor scores) [37]. The details of the base datasets, including the number of subjects and ancestries, are shown in Table 1. No imputation was performed due to the small sample size and the risk of potentially compromising the accuracy of our results. Instead, the PRSs were computed using PRSice-2 software v. 2.3.5 [38] and the formula:

$$\:PRS=\:\sum\:_{i}\frac{{S}_{i}\times\:{C}_{i}}{N}$$

, where *S*_*i*_ is the summary statistic for genetic variant *i* from the base dataset, *C*_*i*_ is the observed effect-allele count of variant *i* in the target dataset, and *N* is the total number of alleles included in the PRS computation. The software was set to generate nine PRSs per risk for different p-value cutoffs from genome-wide significance to no-association, i.e., 5e-08, 0.001, 0.05, 0.1, 0.2, 0.3, 0.4, 0.5 and 1, excluding variants on sex chromosomes. Default software settings were maintained for other parameters, including SNP matching based on reference SNP IDs (rsIDs), a clumping threshold set at *P* ≤ 1, an r-squared value ≥ 0.1, and a maximum distance between SNPs of 250 kb. Linkage disequilibrium was estimated using the target sample set only. The number of variants included in the PRS computation at each p-value threshold for each trait is summarized in Table S1.

### Statistical analyses and bioinformatic environment

Data handling and analyses were conducted using plink 1.9 [39], R version 4.2.2 (2022-10-31) and the tidyverse (v. 2.0.0) package. To assess the associations between PRSs and clinical outcomes, we employed linear regression models. Specifically, we tested the interaction effects of treatment (OSU6162 or placebo) with PRS on 14 clinical measures within a model that included terms for both the individual effects of PRS and treatment, as well as their interaction. The first 10 principal components (PCs) of genome-wide genetic variants from the 48 AD patients were included in all models to account for potential population stratification [40]. The model formula was specified as: Outcome ~ PRS + Treatment + (PRS * Treatment) + PC1 +… + PC10. The clinical measures analyzed were the following: (1) Change % heavy drinking days: The change in the percentage of heavy drinking days from baseline (90 days, Timeline Follow Back) to the 14-day treatment period; (2) Change % drinking days: The change in the percentage of drinking days from baseline (90 days, Timeline Follow Back) to the 14-day treatment period; (3) Change MADRS-S: The change in Montgomery-Åsberg Depression Self-Rating Scale (MADRS-S) scores from baseline to end of treatment (day 15); (4) Change PACS: The change in Penn Alcohol Craving Scale (PACS) scores from baseline to end of treatment (day 15); (5) Change PEth: The change in blood phosphatidylethanol (PEth) levels from baseline to end of treatment (day 15); (6) Study drinks: The total number of drinks consumed during the 14-day treatment period; (7) Study % heavy drinking days: The percentage of heavy drinking days during the 14-day treatment period; (8) Study % drinking days: The percentage of drinking days during the 14-day treatment period; (9–11) Craving (DAQ): The change in Desire for Alcohol Questionnaire (DAQ) scores immediately after the craving session (active cue, neutral cure or priming) compared to baseline; (12–14) Craving (VAS): The change in Visual Analog Scale (VAS) scores immediately after the craving session (active cue, neutral cure or priming) compared to baseline. Multiple testing correction was applied using the Benjamini-Hochberg method to control the False Discovery Rate (FDR), with significance set at FDR < 0.05. The strength and significance of the relationship between PRS and clinical response were also quantified using t-statistics for the estimated regression slopes.

## Results

### Genetic variants in PRS calculations for disorders or traits

As indicated in Table 1, the base GWAS datasets provided summary statistics for over 6 million genetic variants of European or mixed ancestry cohorts across seven disorders or traits, including alcohol use disorder, alcohol consumption (AUDIT-C), problematic alcohol use, drinks per week, depression, and anxiety disorder (binary and continuous). Matching reference SNP IDs between the base GWAS datasets and the full genotyped target cohort (*N* = 405) resulted in 256,268–499,638 variants shared across datasets, representing approximately 47–91% of the 547,984 QC-passed genetic variants from the target dataset. To account for linkage disequilibrium (LD) and retain independent variants, clumping was applied to these shared variants. This step reduced the number of variants used for PRS calculations to 85,590–191,055, depending on the GWAS dataset.

**Table 1:** Summary of base datasets, variant counts, and statistical measures per disorder or trait in each GWAS: this includes the number of subjects and variants in the base GWAS datasets, the number of variants shared between the base and target datasets (“common variants after matching”), and the number of independent variants retained after clumping (performed using PRSice-2). The statistical measures provided by the base datasets, including odds ratios (OR) and beta coefficients, are also shown

Disorder or trait	Base study reference	Ancestry	*N* subjects in base GWAS	Total variants in base GWAS	Common variants after matching^‡^	Variants after clumping	Statistic type
Alcohol use disorder	[33]	European	202,004	6,895,250	256,268	110,604	OR
Alcohol consumption (AUDIT-C)	[33]	European	200,680	6,898,149	256,452	110,779	beta
Problematic alcohol use	[34]	European	435,563	14,068,117	478,784	183,689	beta
Drinks per week	[35]	European	666,978	13,267,983	499,638	191,055	beta
Depression	[36]	Mixed	500,199	8,483,301	448,812	171,188	OR
Anxiety (case-control)	[37]	European	17,310	6,330,995	281,394	86,684	OR
Anxiety (factor score)	[37]	European	18,186	6,306,612	279,053	85,590	beta

^**‡**^Number of variants shared between the base and target datasets, retained for subsequent clumping analysis

AUDIT-C: Alcohol Use Disorders Identification Test-Consumption

GWAS: Genome-Wide Association Study

OR: Odds ratio

### Genetic liability for anxiety correlates with treatment response to OSU6162

**Fig. 1:**
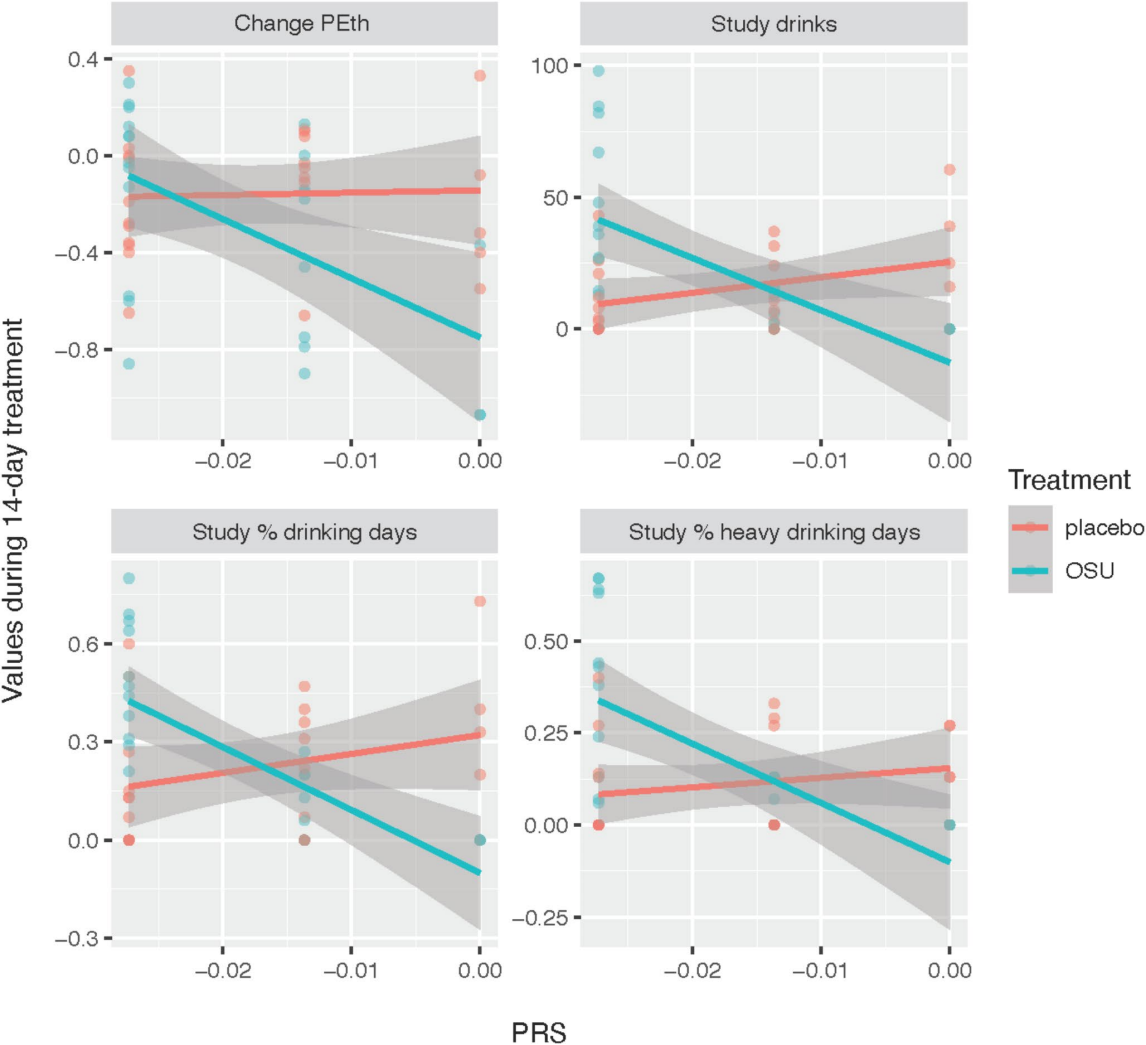
Correlations between anxiety factor PRS at the genome-wide significance threshold and clinical outcomes in alcohol-dependent individuals treated with OSU6162 or placebo. Linear regression models evaluated the interaction between anxiety factor PRS and treatment group (OSU6162 or placebo) over a 14-day period in alcohol-dependent patients (OSU6162: *N* = 24; placebo: *N* = 24). Significance was assessed using t-statistics, corrected for multiple comparisons with the Benjamini-Hochberg method. Significant interactions (FDR < 0.05) were observed at the genome-wide significance PRS threshold (including one SNP, rs698775; Table S1), associated with the number of drinks consumed, percentage of drinking days, and percentage of heavy drinking days (Table 2). A trend (FDR = 0.06) was also observed for changes in blood phosphatidylethanol (PEth) levels. The figure shows correlations between the anxiety factor PRS at the genome-wide significance threshold and clinical outcomes for individual participants, where PRS values correspond to rs698775 genotypes: PRS = 0 (GG), PRS = -0.0136 (AG), and PRS = -0.0273 (AA). Overlapping points may obscure the exact number of individuals in each genotype group (e.g., Study drinks: OSU6162-treated vs. placebo, GG: *N* = 3 vs. *N* = 5; AG: *N* = 9 vs. *N* = 8; AA: *N* = 12 vs. *N* = 11). The t-statistics and corresponding p-values for the slopes of individual outcomes are as follows (OSU6162-treated vs. placebo): Change PEth: -3.05 vs. 0.17, *p* = 0.005 vs. *p* = 0.860; Study drinks: -3.85 vs. 1.89, *P* = 0.00086 vs. *p* = 0.071; Study % drinking days: -4.85 vs. 1.43, *p* = 0.000074 vs. *p* = 0.165; Study % heavy drinking days: -3.83 vs. 0.99, *p* = 0.00089 vs. *p* = 0.329. Data were obtained from a randomized, double-blind, placebo-controlled trial conducted between 2012 and 2013 at the Stockholm Centre for Dependency Disorders, Stockholm, Sweden. The study was approved by the Regional Ethics Committee of Stockholm (Dnr 2011/1707-31/4) and registered in the European Clinical Trials Database (EudraCT; 2011-003133-34). Treatment groups are indicated by color: red for placebo and teal for OSU6162 (OSU). Change PEth: The change in blood phosphatidylethanol (PEth) levels from baseline to end of treatment (day 15); Study drinks: The total number of drinks consumed during the 14-day treatment period; Study % heavy drinking days: The percentage of heavy drinking days during the 14-day treatment period; Study % drinking days: The percentage of drinking days during the 14-day treatment period; FDR, False Discovery Rate; OSU6162, A monoamine stabilizer; PRS, Polygenic Risk Score; SNP, Single Nucleotide Polymorphism

Initial analyses revealed no significant differences in PRSs between the OSU6162 and placebo groups (Table S2). Subsequent linear models, adjusted for the first 10 principal components of genome-wide genetic variants to control for population stratification, evaluated interactions between treatment and PRSs across 14 clinical measures with varying p-value thresholds. No significant interactions were found for PRSs related to alcohol use disorder, alcohol consumption (AUDIT-C), problematic alcohol use, drinks per week, depression, and anxiety case-control comparisons (Tables S3-S8). However, significant interactions were found between treatment and the anxiety factor score PRS at the genome-wide significance threshold that included one SNP (rs698775; Table S1). These interactions were significantly associated with reductions in the number of drinks consumed (t = -4.08, FDR = 0.0017; Table 2), the percentage of heavy drinking days (t = -3.50, FDR = 0.0060; Table 2), and the percentage of drinking days (t = -4.10, FDR = 0.0017; Table 2). A trend was also observed for changes in blood phosphatidylethanol (PEth) levels (t = -2.45, FDR = 0.068; Table 2). Figure 1 highlights these findings, illustrating strong negative correlations between the anxiety factor score PRS and drinking metrics in the OSU6162-treated group, as opposed to negligible correlations in the placebo group (t-statistics and corresponding p-values for individual slopes of OSU6162-treatment vs. placebo: Study drinks: -3.85 vs. 1.89, *p* = 0.00086 vs. *p* = 0.071; Study % heavy drinking days: -3.83 vs. 0.99, *p* = 0.00089 vs. *p* = 0.329; Study % drinking days: -4.85 vs. 1.43, *p* = 0.000074 vs. *p* = 0.165; Change PEth: -3.05 vs. 0.17, *p* = 0.005 vs. *p* = 0.860). Figures S1 and S2 display the first four principal components of genome-wide genetic variants, demonstrating that no significant population substructure differences were observed either between treatment groups (OSU6162 and placebo) or across PRS values for the anxiety factor score.

**Table 2:** Impact of anxiety factor score PRS on clinical measures: interaction effects of anxiety factor score PRS with OSU6162 (*N* = 24) or placebo (*N* = 24) on clinical measures, including heavy drinking days, drinking days, MADRS-S, PACS, PEth levels, study drinks, and craving scores (DAQ and VAS)

Clinical measures^‡^	5e-08	0.001	0.05	0.1	0.2	0.3	0.4	0.5	1
Change % heavy drinking days	0.424	0.906	0.157	0.416	0.672	0.805	0.949	0.841	0.95
Change % drinking days	0.111	0.906	0.164	0.416	0.672	0.805	0.949	0.841	0.95
Change MADRS-S	0.675	0.906	0.75	0.938	0.975	0.805	0.949	0.841	0.95
Change PACS	0.32	0.906	0.376	0.562	0.672	0.805	0.949	0.841	0.95
Change PEth	**0.068**	0.906	0.75	0.862	0.939	0.884	0.949	0.847	0.95
Study drinks	0.0017**	0.906	0.493	0.742	0.939	0.805	0.964	0.841	0.95
Study % heavy drinking days	0.0060**	0.906	0.493	0.562	0.939	0.805	0.964	0.841	0.95
Study % drinking days	0.0017**	0.906	0.493	0.562	0.929	0.805	0.949	0.841	0.95
Craving, active cue (DAQ)	0.96	0.906	0.493	0.562	0.939	0.805	0.949	0.847	0.987
Craving, neutral cue (DAQ)	0.568	0.906	0.493	0.562	0.672	0.805	0.949	0.841	0.95
Craving, priming (DAQ)	0.96	0.906	0.676	0.938	0.939	0.884	0.949	0.841	0.95
Craving, active cue (VAS)	0.783	0.906	0.75	0.862	0.929	0.805	0.949	0.841	0.95
Craving neutral cue (VAS)	0.96	0.906	0.555	0.862	0.954	0.875	0.949	0.841	0.95
Craving priming (VAS)	0.783	0.906	0.376	0.416	0.672	0.805	0.949	0.841	0.95

The table header lists the p-value cut-offs for each polygenic risk score (PRS) assessed

The p-values presented in the table result from linear regression analyses of clinical measures based on the interaction between the anxiety factor score PRS and treatment type (OSU6162 or placebo). They have been adjusted for the false discovery rate (FDR) using the Benjamini-Hochberg method to account for multiple testing across 14 clinical measures, with significance denoted as **FDR < 0.01. Data were obtained from a randomized, double-blind, placebo-controlled trial conducted between 2012 and 2013 at the Stockholm Centre for Dependency Disorders, Stockholm, Sweden. The study was approved by the Regional Ethics Committee of Stockholm (Dnr 2011/1707-31/4) and registered in the European Clinical Trials Database (EudraCT; 2011-003133-34)

^‡^Clinical measures: Change % heavy drinking days: The change in the percentage of heavy drinking days from baseline (90 days, Timeline Follow Back) to the 14-day treatment period; Change % drinking days: The change in the percentage of drinking days from baseline (90 days, Timeline Follow Back) to the 14-day treatment period; Change MADRS-S: The change in Montgomery-Åsberg Depression Self-Rating Scale (MADRS-S) scores from baseline to end of treatment (day 15); Change PACS: The change in Penn Alcohol Craving Scale (PACS) scores from baseline to end of treatment (day 15); Change PEth: The change in blood phosphatidylethanol (PEth) levels from baseline to end of treatment (day 15); Study drinks: The total number of drinks consumed during the 14-day treatment period; Study % heavy drinking days: The percentage of heavy drinking days during the 14-day treatment period; Study % drinking days: The percentage of drinking days during the 14-day treatment period; Craving (DAQ): The change in Desire for Alcohol Questionnaire (DAQ) scores immediately after the craving session compared to baseline; Craving (VAS): The change in Visual Analog Scale (VAS) scores immediately after the craving session compared to baseline

## Discussion

The results of the present study suggest that taking into consideration genetic predispositions to AD-comorbid disorders can reveal who would benefit most from OSU6162, a monoamine stabilizer showing promising preclinical and clinical results in AD settings [16–21]. Specifically, in AD individuals treated with OSU6162, we found significant associations between anxiety factor score PRS and several clinical measures of alcohol consumption, such as the number of drinks consumed during the trial, percentage of heavy drinking days (HDD), and percentage of drinking days, including trend changes in blood phosphatidylethanol (PEth) levels pre- and post-treatment. Notably, none of these relationships were observed in the placebo group. These findings point toward the potential anxiolytic effects of OSU6162, suggesting a diminished reliance on alcohol as a coping mechanism in individuals with a genetic predisposition to anxiety [41]. These anxiolytic effects may be driven by the compound’s monoaminergic stabilizing effects [6, 7] and are consistent with rodent studies demonstrating reduced alcohol intake and anxiety-like behaviors with OSU6162 treatment [18]. Interestingly, however, the associations we observed were exclusively linked to the anxiety PRS based on a quantitative factor-score approach, not a binary case-control classification [37]. This emphasizes the intricate nature of anxiety phenotyping and its genetic determinants, as supported by other GWAS findings examining both continuous and binary classifications of anxiety [42]. Importantly, the associations with the anxiety factor score PRS in our study were based on a genome-wide significant threshold involving a single SNP, rs698775. This SNP is in high linkage disequilibrium with the genome-wide significant SNP (rs1067327) reported in the original GWAS dataset [37]. Both SNPs reside within the same locus, which implicates three primary genes, i.e., calmodulin-lysine N-methyltransferase (*CAMKMT*), prolyl endopeptidase like (*PREPL*), and solute carrier family 3 member 1 (*SLC3A1*) [37]. However, a PRS based on a single locus does not capture the polygenic nature of anxiety and may not robustly predict treatment response, warranting larger and more comprehensive GWASs to develop PRSs that can be reliably utilized in clinical practice.

Additional limitations need to be acknowledged in our study: (i) The ancestry composition of our cohort was characterized as 85–90% European based solely on clinical impressions and not by genetic testing. As such, there remains some uncertainty regarding the ancestry of the sample, which could limit the generalizability of our findings to other populations. Nonetheless, to address potential confounding due to population structure, we included the first 10 principal components of genome-wide genetic variants in our models, which should help mitigate bias associated with ancestry differences. (ii) Linkage disequilibrium (LD) was estimated directly within the target sample set without the use of an external LD reference panel. While this approach ensures alignment with the genetic structure of the cohort and preserves statistical power, it may reduce the generalizability of our results. (iii) The retrospective design of our study is characteristic of early research in the field of personalized medicine for AD, where studies often retrospectively examine pharmacogenetic moderators, typically candidate SNPs, yielding results that have not been consistently replicable [43]. This retrospective approach is a major limitation, as evidenced by pharmacogenetic biomarkers that appeared promising but later failed to demonstrate predictive power in prospective studies—such as those involving a functional SNP in the *OPRM1* gene [44–47]. This underscores the imperative for prospective research to substantiate the predictive validity of genetic markers for treatment responses to interventions like OSU6162. (iv) Our study did not incorporate the latest GWAS data for all traits examined. This decision was primarily driven by the ease of access to summary statistics that were available through databases such as dbGAP or directly linked within the studies we referenced. Additionally, the GWAS data used for the anxiety factor score PRS was derived from a relatively small sample size, which may limit the robustness and predictive power of the PRS in our study. Although this was the best available data at the time of our analysis, we recognize that using GWAS data with larger sample sizes could provide more accurate and reliable PRS calculations. Consequently, we recommend that future research in this area consider employing the latest GWAS statistics and PRS tools to ensure the highest accuracy and efficiency in PRS calculations. (v) Our research focused solely on PRS as the predictor of treatment response, without incorporating the multiomics approaches that have been increasingly recognized as pivotal in advancing precision medicine for various diseases including asthma, cancer, infectious diseases, and metabolic disorders [48–51]. To date, only a limited number of studies have employed an omics-based strategy in AD treatment research. Notably, targeted metabolomics has been used to assess the treatment response to acamprosate, offering valuable preliminary insights [52, 53]. Therefore, there is a compelling case for expanding precision medicine initiatives in AD to include pharmacomultiomics, aiming to uncover a broader spectrum of molecular biosignatures that could enhance the prediction of treatment outcomes.

In conclusion, although the findings of the present study are preliminary, they indicate the potential role of anxiety PRS in optimizing the use of monoaminergic stabilizers like OSU6162 for the management of alcohol use disorders. Larger clinical trials of such compounds are warranted, with extended follow-up periods to assess long-term efficacy, and aiming to evaluate more comprehensive genetic data and PRS methodologies, including additional biological markers, to identify treatment responders and shed light on therapeutic mechanisms.

## Electronic supplementary material

Below is the link to the electronic supplementary material.

Supplementary Material 1

## Data Availability

The data underlying this study are not publicly available due to patient privacy regulations. However, they can be made available from the corresponding author upon reasonable request and in compliance with ethical guidelines.

## Abbreviations

AD: Alcohol Dependence
AUD: Alcohol Use Disorder
CAMKMT: Calmodulin-lysine N-methyltransferase
DAQ: Desire for Alcohol Questionnaire
DSM-IV: Diagnostic and Statistical Manual of Mental Disorders, Fourth Edition
EMA: European Medicines Agency
FDA: Food and Drug Administration
FDR: False Discovery Rate
GWAS: Genome-Wide Association Study
HDD: Heavy Drinking Days
MADRS-S: Montgomery-Åsberg Depression Self-Rating Scale
MVP: Million Veteran Program
NAISS: National Academic Infrastructure for Supercomputing in Sweden
NBIS: National Bioinformatics Infrastructure Sweden
OPRM1: Opioid Receptor Mu 1
OR: Odds ratio
OSU6162: A monoamine stabilizer
PACS: Penn Alcohol Craving Scale
PEth: Phosphatidylethanol
PRS: Polygenic Risk Score
PREPL: Prolyl Endopeptidase-Like
QC: Quality Control
rsIDs: Reference SNP IDs
SLC3A1: Solute Carrier Family 3 Member 1
SNP: Single Nucleotide Polymorphism
SNPs: Single Nucleotide Polymorphisms
TLFB: Timeline Follow Back
UPPMAX: Uppsala Multidisciplinary Center for Advanced Computational Science
VA: Veterans Administration
VAS: Visual Analog Scale
