# Supplementary material for: Genetic liability for anxiety and treatment response to the monoamine stabilizer OSU6162 in alcohol dependence: a retrospective secondary analysis
Source: Pharmacol Rep. 2025 Mar 12;77(3):840–9. doi: 10.1007/s43440-025-00707-8 (PMC12066376; doi:10.1007/s43440-025-00707-8)
Supplement: Supplementary file 1 — Supplementary Material 1 [file 43440_2025_707_MOESM1_ESM.docx]

**Supplemental material**

**Table S1.** Number of variants included in PRS calculations at different p-value thresholds

| **Disorder or trait** | **5e-08** | **0.001** | **0.05** | **0.1** | **0.2** | **0.3** | **0.4** | **0.5** | **1** |
| --- | --- | --- | --- | --- | --- | --- | --- | --- | --- |
| Anxiety (case-control) |  | 275 | 9,200 | 16,900 | 29,811 | 40,963 | 50,448 | 58,711 | 86,684 |
| Anxiety (factor score) | 1 | 268 | 9,209 | 16,751 | 29,594 | 40,347 | 49,757 | 57,857 | 85,590 |
| Alcohol use disorder | 9 | 480 | 11,124 | 19,738 | 34,678 | 47,866 | 59,692 | 70,608 | 110,604 |
| Alcohol consumption (AUDIT-C) | 9 | 613 | 11,953 | 20,811 | 36,039 | 49,162 | 60,965 | 71,397 | 110,779 |
| Depression | 49 | 1,590 | 21,181 | 35,200 | 58,657 | 78,619 | 96,374 | 112,417 | 171,188 |
| Drinks per week | 88 | 2,416 | 26,555 | 42,675 | 68,542 | 90,395 | 109,801 | 127,100 | 191,055 |
| Problematic alcohol use | 24 | 1,081 | 20,579 | 35,817 | 61,596 | 83,496 | 102,611 | 119,775 | 183,689 |

The table header lists the p-value cut-offs for each polygenic risk score (PRS) assessed.

AUDIT-C: Alcohol Use Disorder Identification Test-Consumption

**Table S2.** Associations between disorder or trait-PRSs and treatment (OSU6162 and placebo)

| **Disorder or trait** | **5e-08** | **0.001** | **0.05** | **0.1** | **0.2** | **0.3** | **0.4** | **0.5** | **1** |
| --- | --- | --- | --- | --- | --- | --- | --- | --- | --- |
| Alcohol consumption (AUDIT-C) | 0.704 | 0.997 | 0.704 | 0.248 | 0.248 | 0.248 | 0.248 | 0.248 | 0.248 |
| Alcohol use disorder | 0.896 | 0.896 | 0.896 | 0.899 | 0.896 | 0.896 | 0.896 | 0.896 | 0.896 |
| Anxiety (factor score) | 0.744 | 0.744 | 0.744 | 0.744 | 0.828 | 0.744 | 0.744 | 0.744 | 0.744 |
| Depression | 0.666 | 0.351 | 0.666 | 0.666 | 0.666 | 0.666 | 0.666 | 0.666 | 0.666 |
| Drinks per week | 0.432 | 0.599 | 0.432 | 0.432 | 0.432 | 0.432 | 0.432 | 0.432 | 0.432 |
| Problematic alcohol use | 0.573 | 0.361 | 0.361 | 0.361 | 0.361 | 0.361 | 0.361 | 0.361 | 0.361 |
| Anxiety (case-control) |  | 0.958 | 0.958 | 0.958 | 0.958 | 0.958 | 0.958 | 0.958 | 0.958 |

The table header lists the p-value cut-offs for each polygenic risk score (PRS) assessed.

The p-values presented in the table have been adjusted for multiple testing using the Benjamini-Hochberg method to control the false discovery rate (FDR) and are derived from t-test analyses.

AUDIT-C: Alcohol Use Disorder Identification Test-Consumption

**Table S3.** Impact of drinks-per-week PRS on clinical measures: Interaction with OSU6162 or placebo treatment

| **Clinical measures^‡^** | **5e-08** | **0.001** | **0.05** | **0.1** | **0.2** | **0.3** | **0.4** | **0.5** | **1** |
| --- | --- | --- | --- | --- | --- | --- | --- | --- | --- |
| Change % heavy drinking days | 1 | 0.975 | 0.881 | 0.993 | 0.979 | 0.994 | 0.978 | 0.981 | 0.991 |
| Change % drinking days | 1 | 0.975 | 0.903 | 0.993 | 0.979 | 0.994 | 0.978 | 0.981 | 0.991 |
| Change MADRS-S | 1 | 0.975 | 1 | 0.993 | 0.979 | 0.994 | 0.978 | 0.981 | 0.991 |
| Change PACS | 1 | 0.975 | 1 | 0.993 | 0.979 | 0.994 | 0.978 | 0.981 | 0.991 |
| Change PEth | 1 | 0.975 | 0.881 | 0.993 | 0.979 | 0.994 | 0.978 | 0.981 | 0.991 |
| Study drinks | 1 | 0.975 | 0.881 | 0.993 | 0.979 | 0.994 | 0.978 | 0.981 | 0.991 |
| Study % heavy drinking days | 1 | 0.975 | 0.903 | 0.993 | 0.979 | 0.994 | 0.978 | 0.981 | 0.991 |
| Study % drinking days | 1 | 0.975 | 1 | 0.993 | 0.979 | 0.994 | 0.978 | 0.981 | 0.991 |
| Craving, active cue (DAQ) | 1 | 0.975 | 0.881 | 0.993 | 0.979 | 0.994 | 0.978 | 0.981 | 0.991 |
| Craving, neutral cue (DAQ) | 1 | 0.975 | 0.881 | 0.993 | 0.979 | 0.994 | 0.978 | 0.981 | 0.991 |
| Craving, priming (DAQ) | 1 | 0.975 | 1 | 0.993 | 0.979 | 0.994 | 0.978 | 0.981 | 0.991 |
| Craving, active cue (VAS) | 1 | 0.975 | 0.885 | 0.993 | 0.979 | 0.994 | 0.978 | 0.981 | 0.991 |
| Craving neutral cue (VAS) | 1 | 0.975 | 0.881 | 0.993 | 0.979 | 0.994 | 0.978 | 0.981 | 0.991 |
| Craving priming (VAS) | 1 | 0.975 | 0.881 | 0.993 | 0.979 | 0.994 | 0.978 | 0.981 | 0.991 |

The table header lists the p-value cut-offs for each polygenic risk score (PRS) assessed.

The p-values presented in the table result from linear regression analyses of clinical measures based on the interaction between the anxiety factor score PRS and treatment type (OSU6162 or placebo). They have been adjusted for the false discovery rate (FDR) using the Benjamini-Hochberg method to account for multiple testing across 14 clinical measures.

^‡^Clinical measures: Change % heavy drinking days: The change in the percentage of heavy drinking days from baseline (90 days, Timeline Follow Back) to the 14-day treatment period; Change % drinking days: The change in the percentage of drinking days from baseline (90 days, Timeline Follow Back) to the 14-day treatment period; Change MADRS-S: The change in Montgomery-Åsberg Depression Self-Rating Scale (MADRS-S) scores from baseline to end of treatment (day 15); Change PACS: The change in Penn Alcohol Craving Scale (PACS) scores from baseline to end of treatment (day 15); Change PEth: The change in blood phosphatidylethanol (PEth) levels from baseline to end of treatment (day 15); Study drinks: The total number of drinks consumed during the 14-day treatment period; Study % heavy drinking days: The percentage of heavy drinking days during the 14-day treatment period; Study % drinking days: The percentage of drinking days during the 14-day treatment period; Craving (DAQ): The change in Desire for Alcohol Questionnaire (DAQ) scores immediately after the craving session compared to baseline; Craving (VAS): The change in Visual Analog Scale (VAS) scores immediately after the craving session compared to baseline.

**Table S4.** Impact of anxiety (case-control) PRS on clinical measures: Interaction with OSU6162 or placebo treatment

| **Clinical measures^‡^** | **0.001** | **0.05** | **0.1** | **0.2** | **0.3** | **0.4** | **0.5** | **1** |
| --- | --- | --- | --- | --- | --- | --- | --- | --- |
| Change % heavy drinking days | 0.967 | 0.514 | 0.758 | 0.595 | 0.481 | 0.675 | 0.428 | 0.476 |
| Change % drinking days | 0.933 | 0.514 | 0.729 | 0.595 | 0.438 | 0.675 | 0.409 | 0.476 |
| Change MADRS-S | 0.933 | 0.514 | 0.205 | 0.492 | 0.438 | 0.678 | 0.557 | 0.692 |
| Change PACS | 0.715 | 0.514 | 0.507 | 0.636 | 0.438 | 0.675 | 0.557 | 0.692 |
| Change PEth | 0.967 | 0.616 | 0.758 | 0.829 | 0.665 | 0.766 | 0.557 | 0.692 |
| Study drinks | 0.715 | 0.616 | 0.758 | 0.897 | 0.978 | 0.814 | 0.936 | 0.866 |
| Study % heavy drinking days | 0.715 | 0.629 | 0.9 | 0.897 | 0.876 | 0.766 | 0.886 | 0.802 |
| Study % drinking days | 0.715 | 0.54 | 0.758 | 0.88 | 0.876 | 0.814 | 0.735 | 0.812 |
| Craving, active cue (DAQ) | 0.933 | 0.514 | 0.172 | 0.46 | 0.438 | 0.461 | 0.409 | 0.476 |
| Craving, neutral cue (DAQ) | 0.933 | 0.514 | 0.507 | 0.694 | 0.438 | 0.551 | 0.409 | 0.476 |
| Craving, priming (DAQ) | 0.884 | 0.514 | 0.854 | 0.897 | 0.978 | 0.814 | 0.735 | 0.692 |
| Craving, active cue (VAS) | 0.884 | 0.824 | 0.782 | 0.829 | 0.619 | 0.675 | 0.557 | 0.641 |
| Craving neutral cue (VAS) | 0.953 | 0.742 | 0.758 | 0.88 | 0.665 | 0.766 | 0.876 | 0.802 |
| Craving priming (VAS) | 0.967 | 0.514 | 0.758 | 0.897 | 0.876 | 0.766 | 0.59 | 0.692 |

The table header lists the p-value cut-offs for each polygenic risk score (PRS) assessed.

The p-values presented in the table result from linear regression analyses of clinical measures based on the interaction between the anxiety factor score PRS and treatment type (OSU6162 or placebo). They have been adjusted for the false discovery rate (FDR) using the Benjamini-Hochberg method to account for multiple testing across 14 clinical measures.

^‡^Clinical measures: Change % heavy drinking days: The change in the percentage of heavy drinking days from baseline (90 days, Timeline Follow Back) to the 14-day treatment period; Change % drinking days: The change in the percentage of drinking days from baseline (90 days, Timeline Follow Back) to the 14-day treatment period; Change MADRS-S: The change in Montgomery-Åsberg Depression Self-Rating Scale (MADRS-S) scores from baseline to end of treatment (day 15); Change PACS: The change in Penn Alcohol Craving Scale (PACS) scores from baseline to end of treatment (day 15); Change PEth: The change in blood phosphatidylethanol (PEth) levels from baseline to end of treatment (day 15); Study drinks: The total number of drinks consumed during the 14-day treatment period; Study % heavy drinking days: The percentage of heavy drinking days during the 14-day treatment period; Study % drinking days: The percentage of drinking days during the 14-day treatment period; Craving (DAQ): The change in Desire for Alcohol Questionnaire (DAQ) scores immediately after the craving session compared to baseline; Craving (VAS): The change in Visual Analog Scale (VAS) scores immediately after the craving session compared to baseline.

**Table S5.** Impact of alcohol use disorder PRS on clinical measures: Interaction with OSU6162 or placebo treatment

| **Clinical measures^‡^** | **5e-08** | **0.001** | **0.05** | **0.1** | **0.2** | **0.3** | **0.4** | **0.5** | **1** |
| --- | --- | --- | --- | --- | --- | --- | --- | --- | --- |
| Change % heavy drinking days | 0.967 | 0.933 | 0.804 | 0.838 | 0.997 | 0.994 | 0.939 | 0.976 | 0.964 |
| Change % drinking days | 0.967 | 0.933 | 0.804 | 0.838 | 0.997 | 0.994 | 0.939 | 0.976 | 0.9 |
| Change MADRS-S | 0.858 | 0.933 | 0.95 | 0.838 | 0.997 | 0.994 | 0.939 | 0.976 | 0.964 |
| Change PACS | 0.858 | 0.933 | 0.95 | 0.974 | 0.997 | 0.994 | 0.939 | 0.976 | 0.964 |
| Change PEth | 0.858 | 0.979 | 0.845 | 0.896 | 0.997 | 0.994 | 0.939 | 0.976 | 0.964 |
| Study drinks | 0.967 | 0.933 | 0.804 | 0.838 | 0.997 | 0.994 | 0.939 | 0.976 | 0.964 |
| Study % heavy drinking days | 0.967 | 0.933 | 0.804 | 0.838 | 0.997 | 0.994 | 0.939 | 0.976 | 0.964 |
| Study % drinking days | 0.967 | 0.933 | 0.804 | 0.838 | 0.997 | 0.994 | 0.939 | 0.976 | 0.964 |
| Craving, active cue (DAQ) | 0.967 | 0.933 | 0.95 | 0.892 | 0.997 | 0.994 | 0.939 | 0.976 | 0.964 |
| Craving, neutral cue (DAQ) | 0.967 | 0.933 | 0.95 | 0.964 | 0.997 | 0.994 | 0.939 | 0.976 | 0.964 |
| Craving, priming (DAQ) | 0.967 | 0.933 | 0.95 | 0.974 | 0.997 | 0.994 | 0.939 | 0.976 | 0.964 |
| Craving, active cue (VAS) | 0.967 | 0.933 | 0.95 | 0.896 | 0.997 | 0.994 | 0.939 | 0.976 | 0.964 |
| Craving neutral cue (VAS) | 0.967 | 0.933 | 0.904 | 0.838 | 0.997 | 0.994 | 0.939 | 0.976 | 0.964 |
| Craving priming (VAS) | 0.967 | 0.933 | 0.95 | 0.838 | 0.997 | 0.994 | 0.939 | 0.976 | 0.964 |

The table header lists the p-value cut-offs for each polygenic risk score (PRS) assessed.

The p-values presented in the table result from linear regression analyses of clinical measures based on the interaction between the anxiety factor score PRS and treatment type (OSU6162 or placebo). They have been adjusted for the false discovery rate (FDR) using the Benjamini-Hochberg method to account for multiple testing across 14 clinical measures.

^‡^Clinical measures: Change % heavy drinking days: The change in the percentage of heavy drinking days from baseline (90 days, Timeline Follow Back) to the 14-day treatment period; Change % drinking days: The change in the percentage of drinking days from baseline (90 days, Timeline Follow Back) to the 14-day treatment period; Change MADRS-S: The change in Montgomery-Åsberg Depression Self-Rating Scale (MADRS-S) scores from baseline to end of treatment (day 15); Change PACS: The change in Penn Alcohol Craving Scale (PACS) scores from baseline to end of treatment (day 15); Change PEth: The change in blood phosphatidylethanol (PEth) levels from baseline to end of treatment (day 15); Study drinks: The total number of drinks consumed during the 14-day treatment period; Study % heavy drinking days: The percentage of heavy drinking days during the 14-day treatment period; Study % drinking days: The percentage of drinking days during the 14-day treatment period; Craving (DAQ): The change in Desire for Alcohol Questionnaire (DAQ) scores immediately after the craving session compared to baseline; Craving (VAS): The change in Visual Analog Scale (VAS) scores immediately after the craving session compared to baseline.

**Table S6.** Impact of alcohol consumption (AUDIT-C) PRS on clinical measures: Interaction with OSU6162 or placebo treatment

| **Clinical measures^‡^** | **5e-08** | **0.001** | **0.05** | **0.1** | **0.2** | **0.3** | **0.4** | **0.5** | **1** |
| --- | --- | --- | --- | --- | --- | --- | --- | --- | --- |
| Change % heavy drinking days | 0.968 | 0.963 | 0.981 | 0.778 | 0.742 | 0.811 | 0.693 | 0.568 | 0.736 |
| Change % drinking days | 0.968 | 0.963 | 0.851 | 0.778 | 0.526 | 0.586 | 0.635 | 0.487 | 0.677 |
| Change MADRS-S | 0.928 | 0.963 | 0.851 | 0.848 | 0.605 | 0.811 | 0.924 | 0.707 | 0.825 |
| Change PACS | 0.928 | 0.61 | 0.851 | 0.778 | 0.526 | 0.586 | 0.635 | 0.568 | 0.677 |
| Change PEth | 0.968 | 0.963 | 0.851 | 0.778 | 0.526 | 0.586 | 0.507 | 0.299 | 0.677 |
| Study drinks | 0.968 | 0.963 | 0.851 | 0.778 | 0.526 | 0.586 | 0.635 | 0.487 | 0.677 |
| Study % heavy drinking days | 0.968 | 0.963 | 0.851 | 0.778 | 0.526 | 0.586 | 0.635 | 0.487 | 0.677 |
| Study % drinking days | 0.968 | 0.963 | 0.851 | 0.778 | 0.526 | 0.586 | 0.635 | 0.487 | 0.677 |
| Craving, active cue (DAQ) | 0.968 | 0.623 | 0.851 | 0.778 | 0.742 | 0.586 | 0.648 | 0.487 | 0.677 |
| Craving, neutral cue (DAQ) | 0.968 | 0.963 | 0.851 | 0.848 | 0.974 | 0.794 | 0.648 | 0.524 | 0.677 |
| Craving, priming (DAQ) | 0.968 | 0.344 | 0.851 | 0.778 | 0.526 | 0.586 | 0.693 | 0.635 | 0.736 |
| Craving, active cue (VAS) | 0.968 | 0.963 | 0.981 | 0.932 | 0.863 | 0.921 | 0.966 | 0.997 | 0.947 |
| Craving neutral cue (VAS) | 0.968 | 0.963 | 0.851 | 0.944 | 0.742 | 0.811 | 0.966 | 0.955 | 0.871 |
| Craving priming (VAS) | 0.968 | 0.61 | 0.981 | 0.848 | 0.754 | 0.811 | 0.924 | 0.707 | 0.736 |

The table header lists the p-value cut-offs for each polygenic risk score (PRS) assessed.

The p-values presented in the table result from linear regression analyses of clinical measures based on the interaction between the anxiety factor score PRS and treatment type (OSU6162 or placebo). They have been adjusted for the false discovery rate (FDR) using the Benjamini-Hochberg method to account for multiple testing across 14 clinical measures.

^‡^Clinical measures: Change % heavy drinking days: The change in the percentage of heavy drinking days from baseline (90 days, Timeline Follow Back) to the 14-day treatment period; Change % drinking days: The change in the percentage of drinking days from baseline (90 days, Timeline Follow Back) to the 14-day treatment period; Change MADRS-S: The change in Montgomery-Åsberg Depression Self-Rating Scale (MADRS-S) scores from baseline to end of treatment (day 15); Change PACS: The change in Penn Alcohol Craving Scale (PACS) scores from baseline to end of treatment (day 15); Change PEth: The change in blood phosphatidylethanol (PEth) levels from baseline to end of treatment (day 15); Study drinks: The total number of drinks consumed during the 14-day treatment period; Study % heavy drinking days: The percentage of heavy drinking days during the 14-day treatment period; Study % drinking days: The percentage of drinking days during the 14-day treatment period; Craving (DAQ): The change in Desire for Alcohol Questionnaire (DAQ) scores immediately after the craving session compared to baseline; Craving (VAS): The change in Visual Analog Scale (VAS) scores immediately after the craving session compared to baseline.

**Table S7.** Impact of problematic alcohol use PRS on clinical measures: Interaction with OSU6162 or placebo treatment

| **Clinical measures^‡^** | **5e-08** | **0.001** | **0.05** | **0.1** | **0.2** | **0.3** | **0.4** | **0.5** | **1** |
| --- | --- | --- | --- | --- | --- | --- | --- | --- | --- |
| Change % heavy drinking days | 0.605 | 0.933 | 0.541 | 0.599 | 0.343 | 0.648 | 0.321 | 0.313 | 0.358 |
| Change % drinking days | 0.685 | 0.933 | 0.754 | 0.828 | 0.677 | 0.713 | 0.617 | 0.703 | 0.675 |
| Change MADRS-S | 0.605 | 0.933 | 0.42 | 0.906 | 0.713 | 0.788 | 0.923 | 0.971 | 0.879 |
| Change PACS | 0.662 | 0.933 | 0.715 | 0.828 | 0.713 | 0.788 | 0.68 | 0.703 | 0.755 |
| Change PEth | 0.482 | 0.933 | 0.754 | 0.828 | 0.713 | 0.788 | 0.783 | 0.873 | 0.813 |
| Study drinks | 0.662 | 0.933 | 0.42 | 0.535 | 0.343 | 0.648 | 0.321 | 0.313 | 0.314 |
| Study % heavy drinking days | 0.662 | 0.933 | 0.378 | 0.535 | 0.343 | 0.648 | 0.321 | 0.313 | 0.314 |
| Study % drinking days | 0.955 | 0.933 | 0.728 | 0.828 | 0.713 | 0.773 | 0.662 | 0.703 | 0.675 |
| Craving, active cue (DAQ) | 0.823 | 0.933 | 0.384 | 0.554 | 0.713 | 0.778 | 0.617 | 0.635 | 0.675 |
| Craving, neutral cue (DAQ) | 0.662 | 0.933 | 0.18 | 0.535 | 0.713 | 0.773 | 0.68 | 0.773 | 0.813 |
| Craving, priming (DAQ) | 0.662 | 0.933 | 0.368 | 0.535 | 0.601 | 0.713 | 0.321 | 0.313 | 0.358 |
| Craving, active cue (VAS) | 0.662 | 0.933 | 0.355 | 0.535 | 0.343 | 0.675 | 0.321 | 0.313 | 0.314 |
| Craving neutral cue (VAS) | 0.662 | 0.933 | 0.754 | 0.624 | 0.713 | 0.713 | 0.662 | 0.703 | 0.675 |
| Craving priming (VAS) | 0.685 | 0.933 | 0.355 | 0.535 | 0.601 | 0.713 | 0.504 | 0.313 | 0.547 |

The table header lists the p-value cut-offs for each polygenic risk score (PRS) assessed.

The p-values presented in the table result from linear regression analyses of clinical measures based on the interaction between the anxiety factor score PRS and treatment type (OSU6162 or placebo). They have been adjusted for the false discovery rate (FDR) using the Benjamini-Hochberg method to account for multiple testing across 14 clinical measures.

^‡^Clinical measures: Change % heavy drinking days: The change in the percentage of heavy drinking days from baseline (90 days, Timeline Follow Back) to the 14-day treatment period; Change % drinking days: The change in the percentage of drinking days from baseline (90 days, Timeline Follow Back) to the 14-day treatment period; Change MADRS-S: The change in Montgomery-Åsberg Depression Self-Rating Scale (MADRS-S) scores from baseline to end of treatment (day 15); Change PACS: The change in Penn Alcohol Craving Scale (PACS) scores from baseline to end of treatment (day 15); Change PEth: The change in blood phosphatidylethanol (PEth) levels from baseline to end of treatment (day 15); Study drinks: The total number of drinks consumed during the 14-day treatment period; Study % heavy drinking days: The percentage of heavy drinking days during the 14-day treatment period; Study % drinking days: The percentage of drinking days during the 14-day treatment period; Craving (DAQ): The change in Desire for Alcohol Questionnaire (DAQ) scores immediately after the craving session compared to baseline; Craving (VAS): The change in Visual Analog Scale (VAS) scores immediately after the craving session compared to baseline.

**Table S8.** Impact of depression PRS on clinical measures: Interaction with OSU6162 or placebo treatment

| **Clinical measures^‡^** | **5e-08** | **0.001** | **0.05** | **0.1** | **0.2** | **0.3** | **0.4** | **0.5** | **1** |
| --- | --- | --- | --- | --- | --- | --- | --- | --- | --- |
| Change % heavy drinking days | 0.859 | 0.429 | 0.421 | 0.221 | 0.383 | 0.474 | 0.287 | 0.377 | 0.34 |
| Change % drinking days | 0.859 | 0.429 | 0.0872 | 0.056 | 0.175 | 0.226 | 0.19 | 0.223 | 0.223 |
| Change MADRS-S | 0.859 | 0.333 | 0.806 | 0.825 | 0.927 | 0.987 | 0.891 | 0.987 | 0.909 |
| Change PACS | 0.859 | 0.429 | 0.748 | 0.825 | 0.927 | 0.8 | 0.74 | 0.684 | 0.593 |
| Change PEth | 0.924 | 0.429 | 0.646 | 0.514 | 0.841 | 0.601 | 0.382 | 0.483 | 0.39 |
| Study drinks | 0.861 | 0.429 | 0.0606 | 0.073 | 0.14 | 0.121 | 0.0711 | 0.0963 | 0.0637 |
| Study % heavy drinking days | 0.859 | 0.429 | 0.05 | 0.056 | 0.127 | 0.121 | 0.0711 | 0.0598 | 0.0525 |
| Study % drinking days | 0.859 | 0.429 | 0.05 | 0.056 | 0.127 | 0.121 | 0.0711 | 0.0598 | 0.0525 |
| Craving, active cue (DAQ) | 0.859 | 0.798 | 0.646 | 0.497 | 0.14 | 0.121 | 0.19 | 0.148 | 0.0949 |
| Craving, neutral cue (DAQ) | 0.924 | 0.539 | 0.841 | 0.825 | 0.927 | 0.8 | 0.742 | 0.684 | 0.604 |
| Craving, priming (DAQ) | 0.924 | 0.724 | 0.646 | 0.442 | 0.153 | 0.121 | 0.0711 | 0.0598 | 0.0525 |
| Craving, active cue (VAS) | 0.859 | 0.76 | 0.841 | 0.825 | 0.927 | 0.987 | 0.891 | 0.987 | 0.909 |
| Craving neutral cue (VAS) | 0.859 | 0.798 | 0.646 | 0.514 | 0.927 | 0.873 | 0.74 | 0.832 | 0.895 |
| Craving priming (VAS) | 0.859 | 0.944 | 0.841 | 0.825 | 0.927 | 0.8 | 0.74 | 0.684 | 0.58 |

The table header lists the p-value cut-offs for each polygenic risk score (PRS) assessed.

The p-values presented in the table result from linear regression analyses of clinical measures based on the interaction between the anxiety factor score PRS and treatment type (OSU6162 or placebo). They have been adjusted for the false discovery rate (FDR) using the Benjamini-Hochberg method to account for multiple testing across 14 clinical measures, with significance denoted as *p<0.05.

^‡^Clinical measures: Change % heavy drinking days: The change in the percentage of heavy drinking days from baseline (90 days, Timeline Follow Back) to the 14-day treatment period; Change % drinking days: The change in the percentage of drinking days from baseline (90 days, Timeline Follow Back) to the 14-day treatment period; Change MADRS-S: The change in Montgomery-Åsberg Depression Self-Rating Scale (MADRS-S) scores from baseline to end of treatment (day 15); Change PACS: The change in Penn Alcohol Craving Scale (PACS) scores from baseline to end of treatment (day 15); Change PEth: The change in blood phosphatidylethanol (PEth) levels from baseline to end of treatment (day 15); Study drinks: The total number of drinks consumed during the 14-day treatment period; Study % heavy drinking days: The percentage of heavy drinking days during the 14-day treatment period; Study % drinking days: The percentage of drinking days during the 14-day treatment period; Craving (DAQ): The change in Desire for Alcohol Questionnaire (DAQ) scores immediately after the craving session compared to baseline; Craving (VAS): The change in Visual Analog Scale (VAS) scores immediately after the craving session compared to baseline.

**Figure S1**

**Fig. S1.** Scatterplots of the first four principal components of genome-wide genetic variants, with points coloured by the polygenic risk score (PRS) for anxiety (factor score) calculated at the genome-wide significance threshold (P < 5e-08).

**Figure S2**

**Fig. S2.** Scatterplots of the first four principal components of genome-wide genetic variants, with points colored by treatment group (OSU6162 or placebo).
